# Investigation of MALDI-TOF Mass Spectrometry for Assessing the Molecular Diversity of *Campylobacter jejuni* and Comparison with MLST and cgMLST: A Luxembourg One-Health Study

**DOI:** 10.3390/diagnostics11111949

**Published:** 2021-10-20

**Authors:** Maureen Feucherolles, Morgane Nennig, Sören L. Becker, Delphine Martiny, Serge Losch, Christian Penny, Henry-Michel Cauchie, Catherine Ragimbeau

**Affiliations:** 1 Environmental Research and Innovation (ERIN) Department, Luxembourg Institute of Science and Technology, L-4422 Belvaux, Luxembourg; cpenny@chd.lu; 2 Epidemiology and Microbial Genomics, Laboratoire National de Santé, L-3555 Dudelange, Luxembourg; Morgane.Nennig@lns.etat.lu (M.N.); catherine.ragimbeau@lns.etat.lu (C.R.); 3 Institute of Medical Microbiology and Hygiene, Saarland University, 66421 Homburg, Germany; Soeren.Becker@uks.eu; 4 Swiss Tropical and Public Health Institute, CH-4002 Basel, Switzerland; 5 University of Basel, CH-4003 Basel, Switzerland; 6 National Reference Centre for Campylobacter, Centre Hospitalier Universitaire Saint-Pierre, Université Libre de Bruxelles (ULB), 1000 Brussels, Belgium; delphine.martiny@lhub-ulb.be; 7 Laboratoire de Médecine Vétérinaire de l’Etat, L-3555 Dudelange, Luxembourg; serge.losch@asv.etat.lu; 8 Cellule Scientifique, Chambre des Députés du Grand-Duché de Luxembourg, L-1728 Luxembourg, Luxembourg

**Keywords:** *Campylobacter*, MALDI-TOF MS, subtyping, MLST, cgMLST, machine learning

## Abstract

There is a need for active molecular surveillance of human and veterinary *Campylobacter* infections. However, sequencing of all isolates is associated with high costs and a considerable workload. Thus, there is a need for a straightforward complementary tool to prioritize isolates to sequence. In this study, we proposed to investigate the ability of MALDI-TOF MS to pre-screen *C. jejuni* genetic diversity in comparison to MLST and cgMLST. A panel of 126 isolates, with 10 clonal complexes (CC), 21 sequence types (ST) and 42 different complex types (CT) determined by the SeqSphere+ cgMLST, were analysed by a MALDI Biotyper, resulting into one average spectra per isolate. Concordance and discriminating ability were evaluated based on protein profiles and different cut-offs. A random forest algorithm was trained to predict STs. With a 94% similarity cut-off, an AWC of 1.000, 0.933 and 0.851 was obtained for MLST_CC_, MLST_ST_ and cgMLST profile, respectively. The random forest classifier showed a sensitivity and specificity up to 97.5% to predict four different STs. Protein profiles allowed to predict *C. jejuni* CCs, STs and CTs at 100%, 93% and 85%, respectively. Machine learning and MALDI-TOF MS could be a fast and inexpensive complementary tool to give an early signal of recurrent *C. jejuni* on a routine basis.

## 1. Introduction

*Campylobacter* spp. was recognized as an important human pathogen in the 1970s even if it had been previously described at the end of the 19th century by Escherich in the colons of children [1]. It has emerged as being the main cause of enteritis in humans and the most common foodborne bacterial zoonosis, superseding *Salmonella* spp. infections worldwide. Since 2005, campylobacteriosis is the most prevalent bacterial zoonosis in Europe with an underestimated incidence of 59.7 per 100,000 population in 2019 [2]. It is frequently mentioned as an important health and economic burden [3], which represented 7.5 million disability-adjusted life years (DALYs) in the 2010 Global Burden of Disease Study [4]. According to the European Food Safety Authority (EFSA) and European Centre for Disease Prevention and Control (ECDC) 2019 zoonoses report, *C. jejuni* represented 83.1% of the confirmed cases of campylobacteriosis in Europe [2]. Therefore, *C. jejuni* plays a key-role in the overall campylobacteriosis cases.

The genomic surveillance of *C. jejuni* infections is only applied in few European countries [5], despite the proven applicability of advanced molecular methods (e.g., next generation sequencing (NGS)) in routine surveillance [6] and following standard protocols (cf. ISO/DIS 23418 standard under development [7]). On the other hand, EFSA will request the use of whole genome sequencing (WGS) for the harmonisation of the monitoring of antimicrobial resistances in food-producing animals and derived meat by 2026 [8]. Driven by a high incidence over the last decade (i.e., 103.8 per 100,000 inhabitants in 2018), Luxembourg has the molecular monitoring of *Campylobacter* stemming from patients, food, animal reservoirs and environmental samples at a national level [9,10,11]. 

Multi-locus sequence typing (MLST) consists of the analysis of internal fragments of seven housekeeping genes, i.e., *asp*A, *gln*A, *glt*A, *gly*A, *pgm*, *tkt*, *unc*A, resulting in an allelic profile. It was the first proposed and widely used “gene-by-gene” method to classify *Campylobacter* isolates into genotypes, revealing an unexpected semi-clonal population structure through its application [12,13]. A unique sequence type (ST) is assigned to a unique combination of alleles. Alternatively, core-genome MLST (cgMLST), which is an improvement of the MLST, contains a hundred to a thousand of core genes, and therefore show a higher discriminatory power than classical MLST typing scheme. Genomics may determine the clonal relationships between isolates with an unprecedented resolution [14,15]. For *C. jejuni*, three main cgMLST typing schemes were developed, i.e., the Oxford scheme with 1343 loci [16]; the one from Ridom SeqSphere+ software (Ridom GmbH, Münster, Germany) with 637 loci, resulting into complex type (CT), and the INNUENDO scheme with 678 loci [17,18]. All showed high concordance when compared together [19]. However, the existence of different typing methods with different typing schemes underlines there is not a unique standard subtyping methodology for *Campylobacter* [20] and the lack of a common nomenclature.

Over the past 15 years, the diagnostics field took a new turn with the development of cheaper molecular tests, such as DNA-based assays (e.g., polymerase chain reaction) or proteomic analyses. Matrix-assisted laser desorption/ionization time of flight (MALDI-TOF) mass spectrometry (MS), based on protein fingerprints, has become a popular technique in clinical microbiology and is now the reference method for the fast, reliable, and cost-efficient identification of microorganisms. On one hand, it has been successfully applied in routine for the identification of various microorganisms at the species level including aerobic and anaerobic bacteria, mycobacteria and yeasts including mycobacterium, and fungi in diagnostics [21,22,23]. On the other hand, researches in taxonomy usefulness extended to a wider range of organisms have suggested new perspectives, such as for helminths [24,25,26], for ectoparasites (e.g., ticks, fleas, mosquitoes), protozoa, and even more recently for the screening of the SARS-CoV-2 [27,28,29,30]. Further, MALDI-TOF MS has been used for other research proposes such as antimicrobial resistance screening [31,32,33].

Several reports highlighted the ability of MALDI-TOF MS to subtype different microorganisms at the sequence type (ST) level and even single clones, by the identification of specific peaks [34,35,36]. For example, Meng et al. (2019) investigated the molecular epidemiology of carbapenem-resistant *Klebsiella pneumoniae* by using MALDI-TOF MS and MLST [37]. Giacometti et al. (2018) evaluated the ability of MALDI-TOF MS to characterize *Arcobacter butzleri* strains according their peak patterns and performed a comparative analysis with MLST and pulsed field gel electrophoresis (PFGE) [38]. Along the same line, several reports showed it was possible to differentiate allelic isoforms within *Campylobacter* spp. spectra [39,40,41,42]. Indeed, thanks to the presence of specific peak shift in the 2–20 kDa range, Zaunter and colleagues developed the mass spectrometry-based phyloproteomics (MSPP), with the creation of a scheme including 14 different biomarkers, enabling the subtyping as well as sub-grouping *C. jejuni* ssp. *doylei* [43]. Nevertheless, most of the cited studies rely on empirical observations or statistical methods for the identification of discriminatory peaks.

Important breakthroughs have been possible thanks to the optimization of analysis of mass spectra with machine learning methods [44]. Conventional mass spectra analysis relies on few features, such as peak height or area under the peak, whereas machine learning algorithms are able to extract and analyse useful information which are embedded in mass spectra, that conventional approaches cannot detect, making it a powerful and promising tool for further applications [44]. Studies combining mass spectrometry and machine learning algorithms are focusing on antimicrobial susceptibility testing in both bacteria and fungi [45,46]; on the differentiation of close related species (e.g., *Escherichia coli* and *Shigella* spp.) [47] and on serotyping [48]. Moreover, such prediction approach has also been employed for the differentiation of clonal lineages of relevant clinical pathogens, such as methicillin-resistant *Staphylococcus aureus* [49,50].

As highlighted earlier, campylobacteriosis is the most reported bacterial zoonosis worldwide. The actual problem with *Campylobacter* surveillance is the numerous amounts of isolates to sequence daily and its generated high cost. While it is already implemented in routine at the Luxembourg’s reference national center level, many European member states and routine laboratories may not be able to assume such routine for financial and staff reasons. Thus, there is a need of a straightforward and faster alternative/complementary tool to current surveillance methods. Such a tool should give an early signal putting forward related cases of campylobacteriosis, and hence making easier strain sorting for sequencing. Therefore, the aim of this study was to figure out whether, the widely implemented MALDI-TOF MS, best-known for its analysis of speed and cost-efficiency, was able to assess the genetic diversity and the population structure of a selected Luxembourg One-Health *C. jejuni* collection, congruently to genomic classification by MLST and cgMLST. Additionally, an exploration of the potential of machine learning for making subtyping swift and automatic is also considered to look over its potential for future routine application.

## 2. Materials and Methods

### 2.1. Collection 

A set of 126 strains of *C. jejuni* was selected from the national molecular monitoring program, carried out between 2005 and 2021, in Luxembourg. Strains were isolated from food samples (e.g., bovine, ovine and poultry) (*n* = 41), human (*n* = 83) and environment (e.g., surface water, *n* = 2) sources. All strains were subjected to WGS and characterized by MLST (*n* = 7 loci) and cgMLST (*n* = 637 loci) by using the Ridom SeqSphere+ software platform (Ridom GmbH, Münster, Germany) resulting in 10 Clonal Complex (CC, MLST), 21 Sequence Type (ST, MLST) and 42 Complex Type (CT, cgMLST). 

Among these isolates, a total of 74 were identified in a previous study, Nennig et al. (2021), as belonging to four different lineages, i.e., A (*n* = 34), B (*n* = 15), C (*n* = 15) and D (*n* = 10), based on their ST-*gyr*A-*por*A combination and their frequency in human infection over time. Three clones, defined as a set of independent isolated bacteria with similar genotypic characteristic, were identified in isolates (Lineage A (*n* = 31), B (*n* = 12) and D (*n* = 9)), by complete genomic analysis, including 3 cgMLST schemes and whole genome MLST (wgMLST). Concerning the rest of the collection, no other clones were identified. Details of the collection are available in the Supplementary File S1.

### 2.2. MALDI-TOF MS Analysis

#### 2.2.1. Sample Preparation

Each strain was streaked on chocolate agar plates (Thermo Scientific, Waltham, MA, USA) with a loopful using a −80 °C stock suspension stored in FBP medium complemented with *Campylobacter* growth supplement (Thermo Scientific, Waltham, MA, USA), and incubated for 48 ± 2 h at 42 °C under micro-aerobic conditions (5% O_2_, 10% CO_2_, 85% N_2_) using CampyGen 2.5 L gas packs (Thermo Scientific, Waltham, MA, USA). 

For each biological assay, a standardized ethanol/acetonitrile protein-based extraction was performed. Each strain was suspended in 300 µL milliQ water and 900 µL absolute ethanol (Merck, Darmstadt, Germany). The mix was centrifuged for 2 min and the residual ethanol supernatant was discarded. A total of 25 µL of both 70% formic acid (Merck, Darmstadt, Germany) and acetonitrile (Merck, Darmstadt, Germany) were added up to the dry pellet. A final centrifugation was performed, and then 1 µL of supernatant was spotted thrice onto a one-use MALDI Biotarget 96 targets (Bruker Daltonics GmbH, Bremen, Germany). As soon as the samples were dried, the spots were overlaid with 1 µL of portioned HCCA matrix solution (Bruker Daltonics GmbH, Bremen, Germany) prepared with standardized acetonitrile (50% *v*/*v*), water (47.5%) and trifluoroacetic acid (2.5%) solution (Sigma-Aldrich, Saint Louis, MO, USA). Bruker Bacterial Test Standard (BTS), which is a mix of *Escherichia coli* proteins supplemented with RNAse A and myoglobin, was used for external calibration of the apparatus.

#### 2.2.2. Data Acquisition 

MALDI-TOF MS analyses were fulfilled with a Biotyper Microflex LT/SH (Bruker Daltonics GmbH, Bremen, Germany) by using the AutoXecute acquisition method (MBT_AutoX) in FlexControl software v3.4., with a 2–20 kDa mass-to-charge ratio (m/z) range in a positive linear mode. Before measurement, the system was calibrated using the automatic calibration feature with the BTS. For each sample spot, an automatic acquisition with 240 laser shots was performed.

The workflow was performed on three different days (reproducibility) with three technical replicates on the same day (repeatability), resulting in nine spectra per isolate.

#### 2.2.3. Mass Spectra Analysis

Spectra were uploaded on FlexAnalysis v3.0 (Bruker Daltonics GmbH, Bremen, Germany) and an internal calibration was carried out on the 4365.00 m/z peak, which is shared by all samples and the BTS, with no shift observed in *C. jejuni* [43]. Then, mass spectra were converted into mzML files and imported into BioNumerics v7.6 software platform (BioMérieux, Craponne, France). Spectra were pre-processed using the strict program template (rolling disc: 50 points, CWT noise, Kaiser window: 20 points/beta = 10, rolling disc: 200 points) with a sound-to-noise ratio threshold of 20. Spectra of technical replicates were summarized to create an average spectra or main spectra profile (MSP) per isolate. 

MSP were used to calculate an unweighted pair group method with arithmetic mean (UPGMA) dendrogram using a curve based ranked Pearson correlation similarity coefficient, as it is less sensitive to outliers. The corresponding ST has been indicated using a colour code, a same ST can be classified in different CTs. Three cut-offs of, 92%, 93% and 94% of similarity, have been selected to have a close number of clusters than CC, ST and CT respectively defined by cgMLST analysis. Threshold choice was made by investigating the similarity-cluster size plot (Supplementary File S2).

For each similarity-based cluster identified, a MALDI-profile number was attributed to each MSP, allowing partitions mapping. Specific peak matching parameters were applied: constant tolerance: 1 m/z, linear tolerance: 300 ppm, peak detection rate: 20%, on all peak classes. Therefore, peaks within this range were appraised to belong to the same peak group. 

### 2.3. Typing Methods Concordance

Concordance and discrimination power of the three typing methods, i.e., MLST, cgMLST and MALDI-TOF MS, were estimated by using the adjusted Wallace coefficient (AWC) [51] and the Simpson’s index of diversity (SID) [52], respectively, using the online comparing partitions tool (http://www.comparingpartitions.info/ accessed on 10 August 2021). AWC is the probability that two strains with the same typing profile are classified together through a given method while using another typing method. SID translates the probability that two different strains will be placed into different typing groups. Both values were estimated with their 95% confidence interval (CI).

### 2.4. Machine Learning Approach

#### 2.4.1. Data Pre-Processing

A character table showing peaks intensity values of the peak matching table was exported into a csv. file and was labelled with the respective ST profiles. ST groups with less than 5 representatives were excluded from this part of the study, resulting into 91 MSPs to analyse. Such criteria of selection have been applied to avoid having less than two representatives during the validation phase. All features were standardized using a min–max scaler, which transformed values into the (0, 1) range, where 0 and 1 will be the minimum and the maximum respectively. Such a step is performed as variables that are measured at different scales may not contribute equally to the model fitting, thus creating a bias in the end. MSPs were randomly split into 80% (*n* = 63 MSPs) training and 20% (*n* = 28 MSPs) test datasets, with a stratification based on their ST. The training dataset is implemented to build up a prediction model, while the test panel is used to validate the trained model.

#### 2.4.2. Prediction Models and Evaluation

A random forest model was trained. A 10-fold cross validation was performed to establish the overall accuracy of each model. K-fold cross validation is a resampling method which estimates the performance of the machine learning model. Once the best performing model has been chosen based on metrics described below, performance on data not yet seen by the model, has been carried out by using the test dataset.

#### 2.4.3. Evaluation Metrics

To evaluate the different and final models, a multiclass confusion matrix was carried out. Different metrics for multiclass classification, such as the model’s precision, recall, macro F1-score and balanced accuracy will be calculated as they are not affected by the number of cases of each class in case of an imbalanced dataset [53]. The precision, also called positive predictive value, reflects the reliability of the model when a positive value is predicted. The recall, also called sensitivity, measures how the model can find all true positive values. The accuracy computes how much the model is correctly predicting on the entire dataset. In the case of a balanced accuracy, a mean of the recall for each class is calculated, therefore, every class has the same importance and weight. The F1-score measures the model accuracy by aggregating the precision and the recall into a harmonic mean, where 1 is the best score whereas 0 is the worst. In case of a macro F1-score, classes with different size are equally weighted. 

#### 2.4.4. Retro-Engineering

To go further in the analysis, algorithms such as decision tree (DT) based on the dataset, showed features of importance, meaning the peaks that the algorithm used to classify spectra based on their ST. DT is a widely used supervised machine learning algorithm, represented under the shape of a tree with nodes and branches. Here, each branch depends on the intensity of each mass spectra peak. Inside each node, information about the feature name, impurity, i.e., the Gini ratio, the number of isolates per nodes and categories, and the class gave at each node. The Gini index measures the probability of an isolate to be wrongly classified when it is randomly chosen where 0 denotes that all isolates belong to a certain class and 1 denotes all elements are randomly distributed. In biology such algorithms may be helpful to potentially understand biological mechanisms. In our case, it will be to understand which protein may be associated with a specific MLST or cgMLST profiles. All biomarkers retained by the algorithm were checked on Uniprot (https://www.uniprot.org/ accessed on 13 August 2021) according to their mass in Da. Average theoretical masses were calculated using the online Expasy portal tool (http://web.expasy.org/compute_pi/ accessed on 13 August 2021) based on Uniprot amino acid sequence.

The machine learning workflows were carried out using Python programming language (v3.7.6) and the Scikit-learn package (v0.22.1) in Jupyter Notebook (v6.0.3). Detailed information on data analysis is shown in Supplementary File S3.

## 3. Results

### 3.1. Spectra Quality

A total of 1134 spectra acquired after an ethanol/acetonitrile extraction were identified by the Bruker BDAL database (*n* = 8468 spectra) on MBT compass explorer (v4.1). All isolates were identified as *C. jejuni* with a score average ≥ 2.00 and all BTS were identified as *E. coli* with a score average ≥ 2.00. A score of ≥ 2.30 represents reliable species level identification; score 2.00–2.29, probable species level identification; score 1.70–1.90, probable genus level identification, and score ≤ 1.70 is considered an unreliable identification. Then, the reproducibility of MSPs based on spectra similarity, using a Pearson correlation coefficient, was established. Inter-spectra similarity average was 85.6% with a standard deviation of 12.9%. 

### 3.2. Classification

**Figure 1:**
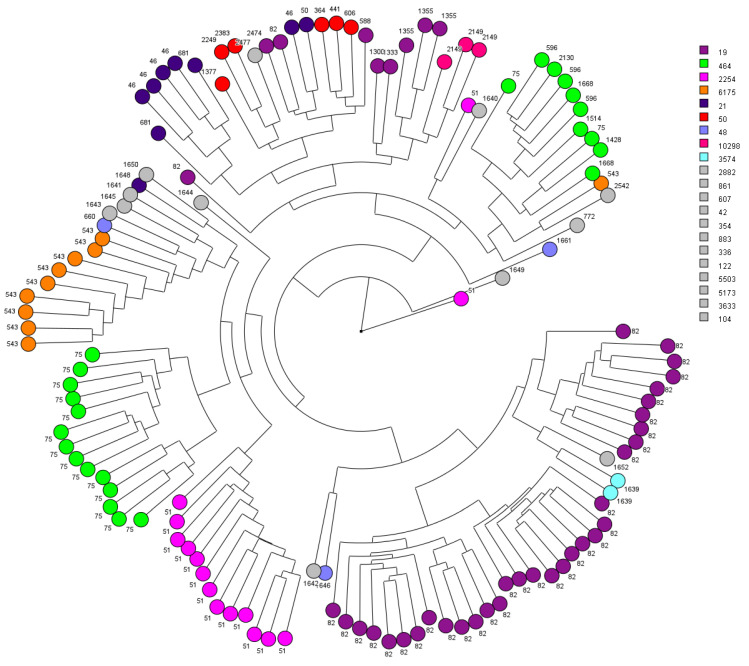
Circular top-score UPGMA dendrogram (ranked Pearson correlation coefficient) based on MSPs grouped by their sequence type (ST) profiles using logarithmic scaling. The whole spectra have been used to compute the figure (*n* = 91 peaks). Colours represents specific ST and outside numbers are CT profiles. Gray colour represents groups with one representant per ST.

As a first step, the clustering of MSPs was investigated in relation to their ST and CT determined by cgMLST. A dendrogram was generated using the 126 MSPs (*n* = 1134 spectra) with all peak classes (*n* = 91 peaks) (Figure 1). Consequently, strains associated to ST-464 (*n* = 24) were subdivided into two main clusters, one grouping a majority of CT-75 (*n* = 14/16) and another one grouping other CTs such as CT-596 or CT-1514. Overall, several isolates which were clustered together belongs to the same ST. For example, 86.7% of ST-2254 (*n* = 13/15), 90.0% ST-6175 (*n* = 9/10), 100.0% ST-10298 (*n* = 3/3) and 100.0% ST-3574 (*n* = 2/2) were clustered together.

Then similarity threshold according to the number of CC, ST and CT’s clusters were selected. Each MSP, sharing more than 92%, 93% and 94% similarity, were assigned to a same MALDI profile number. This resulted in 12, 20 and 40 distinct clusters. A partition mapping has been carried out for STs and CTs grouped by their MALDI profiles, resulting in a contingency table available in Supplementary File S4. The discriminatory ability between proteomics and genomics methods was tested. For this, a SID was calculated for the three methods, i.e., MALDI-TOF MS, including the three different similarity thresholds, cgMLST and MLST from the SeqSphere+ software platform (Table 1).

SID of MALDI-TOF profiles with a threshold of 92%, 93% and 94% were compared to CC, ST, and CT respectively. On one hand, mass spectrometry had a significant higher discriminatory power than MLST_CC_, i.e., 0.830 versus 0.579 respectively. On the other hand, with a SID of 0.862 and 0.939, mass spectrometry had a similar discriminatory power than MLST_ST_ and cgMLST, with a SID of 0.829 and 0.887.

MALDI-TOF MS profiles (threshold = 94%) were investigated for the three clones, identified in a previous study (Supplementary File S1). Clone belonging to the Lineage A (*n* = 31/34) was represented by four different MALDI-TOF MS profiles: 19 (*n* = 9/31), 20 (*n* = 1/31), 30 (*n* = 1/31), which were specific to the clone, while the MALDI-TOF MS Profile 22 (*n* = 20/31) also referred to three other isolates of the Lineage A. Clone belonging to the Lineage B (*n* = 11/13) was assimilated to three MALDI-TOF MS profiles: 14 (*n* = 1/11), 15 (*n* = 1/11), 40 (*n*= 1/11), which were specific to the clone, while the MALDI-TOF MS Profile 13 (*n* = 9/11) is found in the two other isolates of the Lineage B. Clone belonging to the Lineage D (*n* = 9/10) was linked to four MALDI-TOF MS profiles: 2 (*n* = 1/9), 3 (*n* = 1/9), 34 (*n* = 1/9), which were specific to the clone, while MALDI-TOF MS Profile 1 (*n* = 6/9) also referred to another isolates of the Lineage D. In the end, those MALDI-TOF MS profiles were only found in Lineages A, B and D. As well MALDI-TOF MS profiles 10 (*n* = 8/14), 11 (*n* = 1/14) and 12 (*n* = 5/14) were only linked to Lineage C. Average similarity between specific lineage MALDI-TOF MS profiles was close to the defined cut-off (94%). For example, for Lineage A, when the MALDI-TOF MS Profile 19 is compared to the Profiles 20 and 22, the average similarity was 93.6% and 93.3% respectively. The MALDI-TOF MS Profile 30 was less close to the MALDI-TOF MS Profile 19 (90.0%).

### 3.3. Congruency: Proteomics vs. Genomics

According to previously described results, MALDI-TOF MS spectra may be clustered with spectra related to the same genotype, as defined as specific combination of alleles. Therefore, we looked over for the congruency between proteomics and genomics methods. For this, an AWC has been calculated for the three methods, i.e., MALDI-TOF MS, cgMLST and MLST (ST and CC) from the SeqSphere+ software platform (Table 2) by using MALDI-TOF MS profiles with the three different thresholds, i.e., 92%, 93% and 94%, STs and CTs. Overall, MALDI-TOF MS with a 94% similarity threshold shown a high concordance for both MLST and cgMLST typing scheme.

MALDI-TOF MS profiles with a threshold of 92%, 93% and 94% were compared to CC, ST, and CT respectively. When the threshold was settled according to the CCs, mass spectrometry was able to predict 88.1% of CCs. As well, when the ST’s threshold was applied, mass spectrometry could predict 72.5% of STs. Finally, when the CT’s threshold was settled, MALDI-TOF MS was able to predict 85.1% of CT. Overall if the last threshold (94%) was kept for analysis, MALDI-TOF MS could predict 100.0%, 93.3% and 85.1% of CCs, STs, and CTs, respectively.

### 3.4. Machine Learning for Automatic Attribution of ST

MALDI-TOF MS has a high concordance for the MLST method, so a supervised Machine Learning approach was applied to swiftly predicted STs of unknow spectra. In this context, a total of 91 MSPs were examined, associated to ST-19 (*n* = 42); ST-464 (*n* = 24); ST-2254 (*n* = 15) and ST-6175 (*n* = 10). 

**Figure 2:**
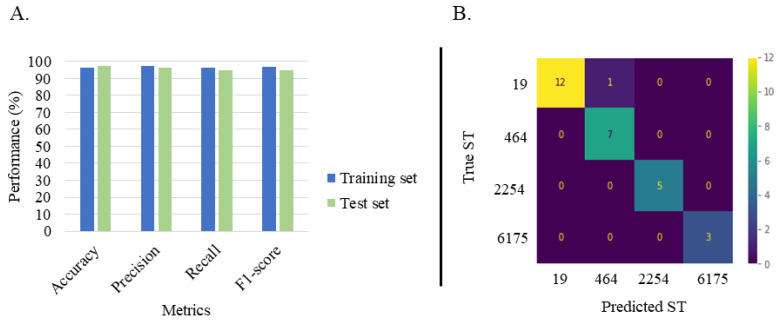
(**A**): Performance metrics for the random forest classifier for both training and test dataset. The horizontal axis was the four metrics balanced accuracy, precision, recall and F1-score, which were averaged over 10-fold cross validations. (**B**): Confusion matrix obtained for the classification of the test set using the trained random forest classifier.

A random forest has been trained and evaluated by using the training dataset. Metrics such as balanced accuracy, precision, recall and F1-score have been calculated with for this purpose. Results are described in Figure 2A. Overall, the trained model had a high performance for the training set (*n* = 63 MSPs), used to build up the prediction model, used to build the model, with values ranging from 96.6% to 97.5%. Therefore, this model was evaluated by performing an external validation by using the test dataset (*n* = 28 MSPs), to appreciate how the model will performed when encountering data, it has not been trained on (Figure 2B). A high performance was obtained for the test set with scores between 95.0% and 97.5%. According to the confusion matrix, the trained random forest classifier could correctly classify studied STs, except for ST-19 where one mismatch was observed. In the end, an average sensitivity and specificity of 98.1% and 100% respectively, were obtained for the current classification.

### 3.5. Features of Importance: Beyond Biomarkers

**Figure 3:**
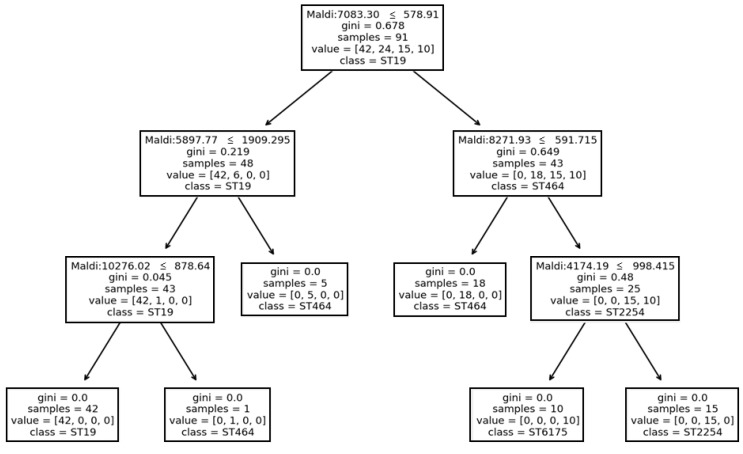
Decision tree trained on the peak matching table with intensity values from the complete dataset (*n* = 91 MSPs) grouped by their ST profiles. Maldi: peak feature; Gini: measures the probability of misclassifying an observation; samples: illustrates how many samples the node contains; value: refers to how many samples at the node fall into each ST category.

Certain machine learning algorithms, such as DT, do not only predict a result based on a probabilistic score, but it may also give a new venue to visualize pattern of features, here proteins, which may be linked to biological mechanisms. In this context, a DT model has been trained on the previous dataset with ST groups with at least five representatives. The related tree was plotted in Figure 3.

**Figure 4:**
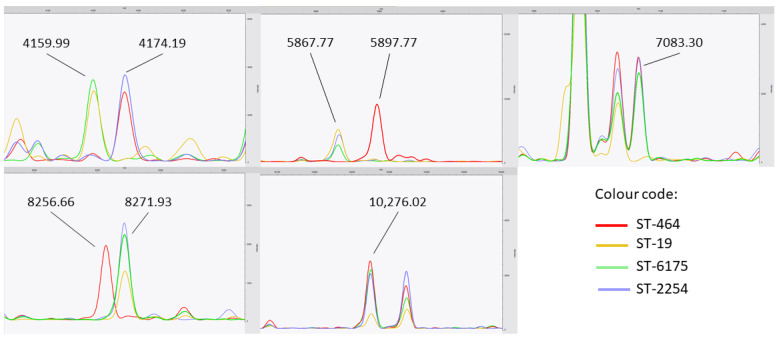
Biomarker mass peak of *C. jejuni* identified by machine learning decision tree algorithm. *X*-axes: mass-to-charge ratio (m/z). *Y*-axes: intensity in arbitrary unit.

In the latter, patterns of protein peaks, based on their intensities, retained by the algorithms for each class could be observed (Figure 4). Overall, for the classification into four different STs, the DT algorithm was considering only to five proteins over the 91 initially identified by the peak matching. A combination of three peaks was enough for the algorithm to distinguish the three different STs: ST-19, ST-2254 and ST-6175. However, the identification of ST-464 seemed a bit trickier with the involvement of several biomarkers, which may be linked the genetic diversity of isolates classified in six different CTs (75, 596, 1428, 1514, 1668 and 2130) (Figure 1). Interestingly, the 4174.19, 5897.77 and 8271.93 Da peaks are associated with 14.20 Da, 30.17 Da, 15.27 Da shifts respectively, while the 7083.30 and 10,276.02 Da peaks were linked to the intensity’s level. Therefore, those proteins are putatively related to the genetic diversity of *C. jejuni*. The Uniprot database has been investigated to give a potential identification of these latter, regardless potential post-translational modifications. Identifications are summarized in the Table 3.

## 4. Discussion

Nowadays, WGS is established as a successful and highly discriminating typing method, providing opportunities for the surveillance and outbreak investigation of foodborne pathogens, such as *Campylobacter* spp. [54]. The main drawback of *Campylobacter* surveillance is the important number of isolates to sequence, due to its status as first bacterial human zoonosis. A high-throughput and cost-efficient method, such as MALDI-TOF MS, could be an efficient pre-screening tool to relevant isolates that warrant further sequencing. By coupling WGS with mass spectrometry, it could increase typing’s ability and therefore, elucidate genotypes circulating in human infections, animal production and environment. The aim of this study was to investigate the ability of MALDI-TOF MS, increasingly implemented in routine laboratories, to assess *C. jejuni* genetic diversity and to compare its congruency to MLST and cgMLST methods as gold standards for epidemiologic surveillance.

The main result of this study was to observe that a mass spectrometry approach on 91 automatically generated peaks had a higher discriminatory power than the classical MLST scheme with seven loci for attribution of CCs (SID_MLST-CC_ = 0.579, SID_MALDI-92%_ = 0.830). However, similar discriminatory power has been found for attribution of STs (SID_MLST-CC_ = 0.829, SID_MALDI-93%_ = 0.862). As well, proteomics was compared to the cgMLST scheme, which is more discriminant than MLST typing methods. The discriminatory ability of MALDI-TOF MS was comparable to SeqSphere+ cgMLST scheme based on 637 loci (SID_cgMLST_ = 0.889, SID_MALDI-94%_ = 0.939). Sequence based methods, such as MLST, are known to reflect the population genetics and where STs are often related to ecological niches [13,55]. As an outlook of the present study, mass spectra should be investigated to check the potential link between protein profile and host specificity, barely described in the literature for *Campylobacter* spp. [56]. Lawton et al. (2018) reported that MALDI-TOF MS was non-congruent to *Campylobacter* clade identified by either 16S rDNA or WGS and therefore unlikely to be useful for assessing genetic relationship among *C. jejuni* isolates [57]. Nevertheless, in the current study high concordance between genomic and proteomic typing methods was found. MALDI-TOF MS could predict 100.0%, 93.3% and 85.1% of CCs, STs and CTs, respectively. To our knowledge this is the first time that this was demonstrated for cgMLST. There are few reports highlighting the ability of MALDI-TOF MS to subtype bacterial species at the ST level. During a *Klebsiella pneumoniae* outbreak in central China, the vast majority of the epidemic ST11 strains were associated with similar MALDI-TOF MS profiles [37]. MALDI-TOF MS was explored for the subtyping of *Arcobacter butzleri* and compared with MLST. MALDI-TOF MS was less discriminant (SID_MLST_ = 0.920, SID_MALDI_ = 0.863) but still comparable to MLST. Therefore, the possibility of subtyping by MALDI-TOF MS displayed variability in performance according to bacterial species. In addition, it may be explained by the quality, pre-processing steps and chosen similarity cut-off, depending on the level of concordance intended, of mass spectra. Indeed in the study by Meng et al. (2019), a similarity cut-off of 70% was applied according to the ST assignment of *K. pneumoniae*, whereas a 93% one has selected for the current study, based on the number of ST clusters. This difference may be explained by the type of extraction, such as the off-plate procedure, used in this study, and the on-plate protocol. Standardisation of protocols for such analysis should be as well essential. Indeed, it has been pointed out that growing medium type and conditions could influence MALDI-TOF MS data and congruence with the PFGE typing method [58]. Additionally, other parameters such as preparation protocol used, duration of incubation, maintenance of the device and so on, could impacted the quality of the spectra and hence the MALDI-TOF MS discriminatory power [59]. Additionally, fastidious growing conditions (e.g., microaerophilia), such as the one encountered for *Campylobacter* spp., may be impacting for the spectra.

Analysis of the decision tree pointed out several protein peaks or biomarkers, which may be associated to specific STs. Since 2011, pioneering studies highlighted the extended application of MALDI-TOF MS as a tool to discriminate several genera (e.g., *Clostridium*, *Salmonella* and *Staphylococcus*) at subgroups level based on specific biomarkers [60,61,62]. The *Campylobacter* community is not an exception to the rule, and several reports bring biomarkers links with allelic profiles to light [39,40,42,43]. In the case of *C. jejuni*, it was already described in the literature that it was possible to discriminate some STs with 14 specific biomarkers while using ethanol/acetonitrile extraction and direct smear deposit based on PCA-dendrograms [39,43]. One biomarker retained in this study was commonly described previously by Zautner and colleagues (e.g., 10,276.02 Da). They ambiguously identified the previous biomarker as a 30S ribosomal protein S18 while it was uncharacterizable in our study. In the same process, the latter authors tentatively tried to identify relevant biomarkers based on the calculated masses ORFs from WGS data. While most of their biomarkers were ribosomal proteins, several current identified proteins are not related to known functions or involved in metabolic pathways encoded by housekeeping genes. However, it is worth to notice that the comparison of biomarkers in both studies is tricky due to the choice of the internal calibration point: recombinant human insulin peak (5808.29 m/z) and the shared BTS peak (4365.00 m/z), in the two studies. Interestingly, the DT algorithm used the shift between the 4159.99 Da and 4174.19 Da peaks to distinguish ST-2254 and ST-6175. It is worth highlighting that isolates from ST-2254 and ST-6175 have different phenotypic behaviour, in term of adhesion and biofilm formation (Nennig et al., manuscript in preparation). The only known protein matching this molecular weight was the flagellin subunit protein FlaA. Combination of MLST with the major outer membrane protein gene (*porA*) and/or with flagellin A gene (*flaA*), called extended MLST, has been widely described in the literature, for the typing of *C. jejuni*, underlining one more time the close similarity between genomics and proteomics methods [9,63,64,65]. Nevertheless, *flaA* is an accessory gene, giving more flexibility to bacteria for environmental adaptation. In addition, *flaA* shows common and highly variable domains, suggesting that this protein alone, could not be considered as a stable biomarker to assess *C. jejuni* genetic diversity [66,67]. As well, no biomarkers retained in this study had a direct link with housekeeping genes classically used for MLST typing. This assessment is not aberrant, as most of these genes produced proteins with a molecular weight exceeding the 2–20 kDa windows (e.g., AspA: 51,765 Da, GlnA: 53,945 Da) used in the study. To go further, a real peptides sequencing should be performed to assess the actual identity and function of each biomarker.

Several reports highlighted the ability of MALDI-TOF MS to classify different *Staphylococcus aureus* clonal lineages with the help of different machine learning models (e.g., supervised neural network, support vector machine (SVM) and genetic algorithm (GA)). Camoez et al. (2016) reported a sensitivity and specificity of 100.0% and 99.1% for the classification of four *S. aureus* CCs, i.e., which is a group compiling close STs and therefore more general than the ST level. While Zhang et al. (2015) described for the assessment of *S. aureus* ST-239, ST-5, ST-59 and ST-45 by using GA, a sensitivity between 81% and 100% and a specificity between 92% and 100%, we observed an overall sensitivity and specificity for four STs of ranging from 98.1% to 100.0%, respectively. Wang et al. (2018) also described close results on same STs by using a SVM model with an accuracy of 86.4%. All previous cited studies support the idea that machine learning and MALDI-TOF MS present obvious advantages for MRSA typing, such as rapidity, accuracy and cost-efficiency in comparison with MLST and it can be carried out at the same time that routine identification of isolates. In addition, such an approach may remove the need of retrospective epidemiological analysis. While cgMLST is the reference method for the surveillance of *Campylobacter* spp., the combination of mass spectrometry and artificial intelligence may be a suitable tool to make a pre-selection of what need to be sequenced. Indeed, it has been shown recently that genetic lineages were frequently identified in human infection over-time in Luxembourg [19]. Using the same lineage isolates of Nennig et al. (2021), we pushed the study at the clone level, confirmed by three cgMLST schemes and by comparing the pangenome (wgMLST). Eleven specific MALDI-TOF profiles have been linked to specific lineages identified over-time in Luxembourg. In addition, several close-related MALDI-TOF profiles where only related to these three clones. Thus, *C. jejuni* protein mass spectra may be enough to make an early detection of these recurring lineages. Machine learning is for supporting decision making process and giving suggestions on possible outcomes that lead research in a specific direction. Machine learning prediction based on MALDI-TOF mass spectra may be a frontline tool to make a preliminary screening of these recurring genotypes and identify related MALDI-TOF profiles. Nevertheless, WGS may still follow to further elucidate molecular details in case of an outbreak as it has been recently described in Denmark [5].

Along the same line, a recent preprint introduced a pipeline using a surveillance system recording routine results from clinical laboratories, among them MALDI-TOF mass spectra identification [68]. The system detected an abnormal increase of *Streptococcus pneumoniae* identification in a short interval of time. More identification than planned were recorded by the system. Spectra responsible of the alert and the other records from previous months were retained for clustering analyses, resulting in two subtrees which may be associated to two epidemiological events. Authors highlighted that such an investigation technique is not for subtyping but helps in detecting a possible suspicion of bacterial species spread and to prevent or slow down possible outbreaks. In summary, combination of MALDI-TOF MS, machine learning and WGS could be valuable tools for accurate epidemiological surveillance of *Campylobacter* and potentially other relevant clinical or foodborne pathogens.

Nevertheless, the present study presents several limitations. Thus, so far, only four different STs were used to build the current model. Therefore, if the latter is used to identify another STs, then it will be misclassified. Additionally, to build the current model only strains from the Luxembourg monitoring program have been used, hence model’s adaptation will be needed, with the implementation of STs depending on the area of utilization. In the case of *Campylobacter* spp., where the question of the possibility of cross-border genotype existing, it may be critical for long-term monitoring, while using MALDI-TOF MS and machine learning only. However, further analysis must be done to include additional STs, to avoid misclassification, for an accurate and robust screening tool. Additionally, all MALDI-TOF MS analyses were carried out by a unique operator the whole study. Therefore, the operator variation has not been established and may affect conclusions [69]. Finally, spectra were investigated after using the standard off-plate protein extraction using ethanol, formic acid and acetonitrile, used to obtain high-resolution spectra. However, such extraction is not straightforward in a routine context. For that reason, further analyses must be carried out to evaluate the ability of MALDI-TOF MS to subtype *C. jejuni* with spectra obtained with on-plate extraction or direct deposit. 

## 5. Conclusions

In the present study our results provide evidence that MALDI-TOF MS could be a valuable tool to swiftly subtype *C. jejuni*. Such applications may be suitable as a cost-efficient alternative to NGS technologies, with several advantages such as rapidness or congruency with genomics methods up to the CC, ST and CT level. For a “universal”, accurate, and early surveillance and integration of routine laboratories, a single mass spectrum analysis could combine several tests into one examination, i.e., species identification, antimicrobial susceptibility screening and the assessment of genetic diversity. However, WGS may still be needed in addition to MALDI-TOF MS to further assess the relatedness between isolates (e.g., source attribution) in case of an outbreak.

## Figures and Tables

**Table 1:** Simpson’s index diversity (CI 95%) for typing schemes comparison.

	Clusters	SID	CI (95%)
Complex Clonal (CC)	10	0.579	0.495–0.664
Sequence Type (ST)	21	0.829	0.785–0.873
Complex Type (CT)	42	0.887	0.849–0.926
MALDI-TOF MS (Cut-off = 92%)	12	0.830	0.800–0.861
MALDI-TOF MS (Cut-off = 93%)	20	0.862	0.828–0.897
MALDI-TOF MS (Cut-off = 94%)	40	0.939	0.918–0.960

**Table 2:** Adjusted Wallace coefficient (CI 95%) for typing schemes comparison.

Adjusted Wallace Coefficient	MLST (CC)	MLST (ST)	cgMLST (CT)	MALDI (94%)	MALDI (93%)	MALDI (92%)
MLST (CC)		0.284 (0.171–0.396)	0.175 (0.079–0.270)	0.090 (0.040–0.140)	0.212 (0.132–0.293)	0.248 (0.179–0.317)
MLST (ST)	1.000 (1.000–1.000)		0.616 (0.474–0.758)	0.297 (0.197–0.396)	0.563 (0.427–0.699)	0.567 (0.447–0.686)
cgMLST (CT)	1.000 (1.000–1.000)	1.000 (1.000–1.000)		0.439 (0.317–0.561)	0.829 (0.703–0.955)	0.824 (0.696–0.951)
MALDI-TOF MS (94%)	1.000 (1.000–1.000)	0.933 (0.916–0.949)	0.851 (0.830–0.872)		1.000 (1.000–1.000)	1.000 (1.000–1.000)
MALDI-TOF MS (93%)	0.965 (0.934–0.996)	0.725 (0.608–0.843)	0.658 (0.551–0.765)	0.410 (0.309–0.511)		1.000 (1.000–1.000)
MALDI-TOF MS (92%)	0.881 (0.830–0.932)	0.572 (0.470–0.673)	0.512 (0.423–0.602)	0.321 (0.236–0.406)	0.783 (0.724–0.841)	

**Table 3:** Putative biomarkers linked to the *Campylobacter* type and their identification through the Uniprot database.

Mass Observed (Da)	Theoretical Average Molecular Weight (Da)	Gene Names	Protein Name	UniProt ID
4159.99	4158.55	APU78_09005	Flagellin subunit protein FlaA	A0A690Z7F7
4174.19	4173.56	CDX23_07240, FQZ36_04085, FV854_03335	Uncharacterized protein	A0A5Z0CYS5
5867.60	5868.02 5867.95 5867.84	FH034_10320 F1576_10330 FDW21_07355	Sulfurtransferase-like selenium metabolism protein YedF Magnesium transporter CorA family protein Motility accessory factor	A0A5C4YC48 A0A698D3Z1 A0A3Z8JXU3
5897.77	5897.85 5895.96	GSG42_09710 FXB36_09400	Polysaccharide deacetylase DNA adenine methylase	A0A7I9U468 A0A7I9S1R5
7083.30	7081.66	B9Q65_09070, E7P40_09640, F0N82_09625, FC283_09220, FW424_09040	Uncharacterized protein	A0A400EER0
8256.66	8256.27 8256.39 8255.77 8256.96	JJD26997_1194 GD714_06815 EJC82_07015 TM42_09010	Conserved domain protein Uncharacterized protein Uncharacterized protein Membrane protein	A7H434 A0A6W1IK17 A0A6C7UKG7 A0A0D7V4A9
8271.93	8270.56 8271.37 8271.52	N/A FW192_09775 B7Q70_09720	Uncharacterized protein Integrase Terminase small subunit	Q4VRA4 A0A7I9QCT5 A0A5T0PDL9
10,276.02	10,276.22 10,276.17 10,274.87	JJD26997_0928 AT778_09125, B7Q70_06195, C3H43_07780, C3H69_07590, C3H86_07890 A2E15_06760	Uncharacterized protein Uncharacterized protein Glycosyltransferase Family 9 protein	A7H3G2 A0A2U0QNA2 A0A5T0CX51

## Data Availability

The MALDI-TOF mass spectra peak matching table is available in Supplementary File S5. Raw MALDI-TOF spectra are available upon request.

## Supplementary Materials

The following are available online at https://www.mdpi.com/article/10.3390/diagnostics11111949/s1. Supplementary File S1—list and details of isolates used for MALDI-TOF mass spectrometry and machine learning analysis. Supplementary File S2—similarity cluster size plot. Supplementary File S3—Python algorithm for machine learning analysis. Supplementary File S4—contingency table for MALDI, ST and CT profiles comparison. Supplementary File S5—mass spectra peak matching table used for machine learning analysis.
