# Supplementary figures and images for: Investigation of MALDI-TOF Mass Spectrometry for Assessing the Molecular Diversity of Campylobacter jejuni and Comparison with MLST and cgMLST: A Luxembourg One-Health Study
Source: Diagnostics (Basel). 2021 Oct 20;11(11):1949. doi: 10.3390/diagnostics11111949 (PMC8621691; doi:10.3390/diagnostics11111949)

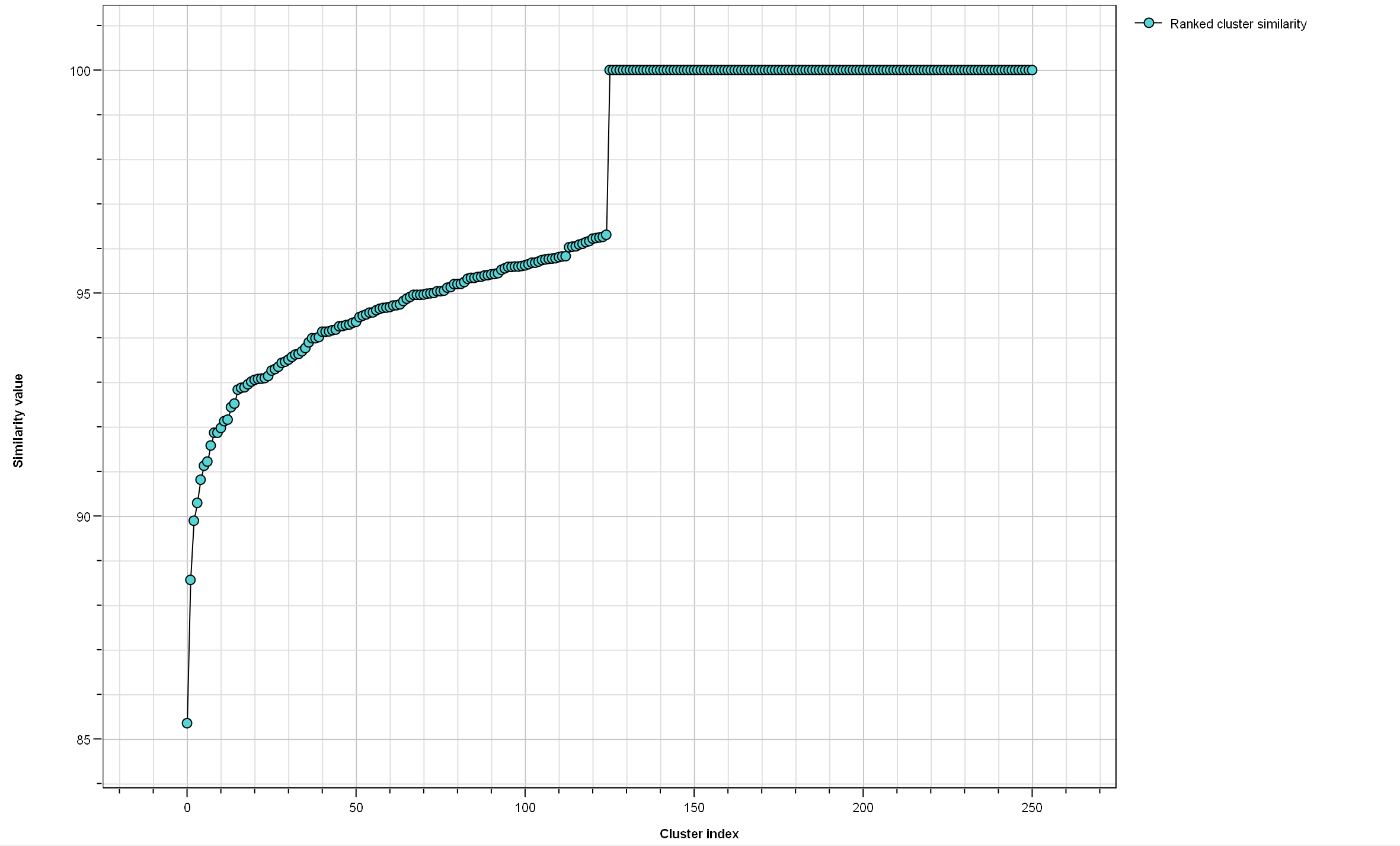

Supplement: Supplementary file 1 [file diagnostics-11-01949-s001.zip › diagnostics-1391958-supplementary/Supplementary file/Supplementary File S2_Similarity_Cluster_size_plot.tiff]
